# First person – Anthony Agudelo

**DOI:** 10.1242/bio.057216

**Published:** 2020-10-21

## Abstract

First Person is a series of interviews with the first authors of a selection of papers published in Biology Open, helping early-career researchers promote themselves alongside their papers. Anthony Agudelo is first author on ‘Age-dependent degeneration of an identified adult leg motor neuron in a *Drosophila* SOD1 model of ALS’, published in BiO. Anthony conducted the research described in this article while an undergraduate Research Assistant in Dr Geoff Stilwell's lab at Rhode Island College, Providence, USA. He is now a Research Assistant in the lab of Dr James Padbury at Rhode Island College, Providence, USA, investigating using computational biology methods to shed light on the pathology of complex diseases.


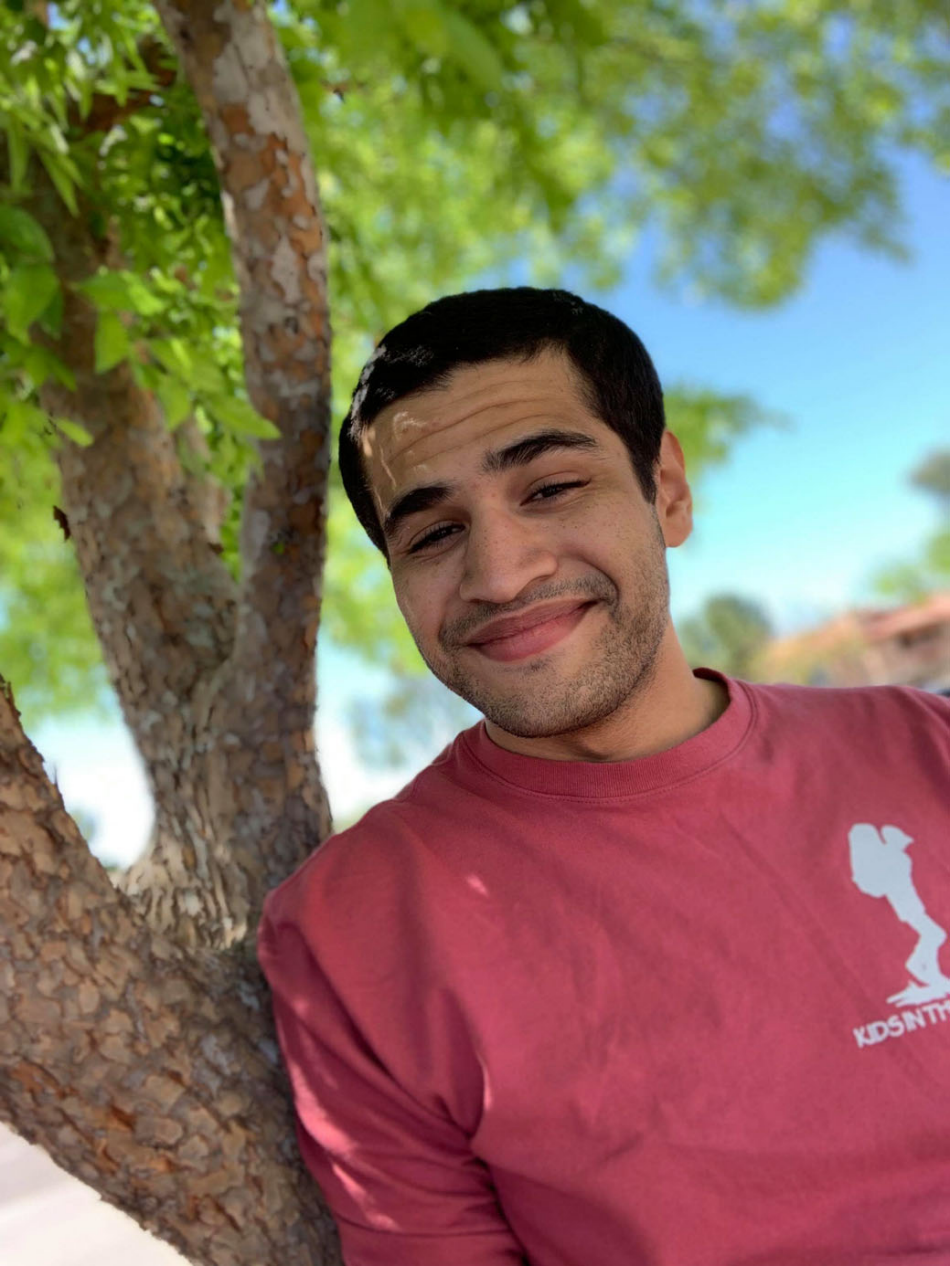


**Anthony Agudelo**

**What is your scientific background and the general focus of your lab?**

I received and BS degree in Biology, BA degree in Mathematics and a BA degree in Chemistry from Rhode Island College. As an undergraduate, my research focused primarily on protein work, utilizing immunohistochemistry and western blot techniques to characterize a *Drosophila melanogaster* model of ALS. Currently I am working in the Padbury lab utilizing computational biology techniques including differential gene expression analysis, pathway analysis, machine learning and deconvolution to better understand two complex diseases, pre-eclampsia and pre-term birth.

**How would you explain the main findings of your paper to non-scientific family and friends?**

Amyotrophic lateral sclerosis or ALS is a progressive neuromuscular disease, individuals diagnosed with ALS will typically only survive 3 to 5 years after being diagnosed. ALS has been shown to be passed on from parent to child in many cases, however, much is still not known about what exactly causes ALS and so that's where the research comes in. Typically when I try to explain the work that I do to my family and friends concisely I will say something along the lines of “we gave fruit flies ALS and now it's my job to see if they show similar characteristics to people with ALS”, the idea being that if we can understand how ALS works in flies then hopefully it can shed light on how the disease works in humans. However, this explanation can be somewhat simplistic and so when I say “we gave fruit flies ALS” what I actually mean is our lab inserted a mutation in a gene called superoxide dismutase (SOD1) that is known to cause ALS when mutated in humans into a fruit fly. What is fascinating and maybe shocking to anyone who is not used to working with animal models of disease is that these fruit flies; animals with an exoskeleton, animals with 1/200,000,000 the mass of a human, animals with the inability to think like humans, actually show very similar ALS-like characteristics to humans when the mutation is inserted into them. The fruit flies with ALS have a much shorter lifespan the average fruit fly; in many cases they also show impairment to their locomotive abilities during the later stages of their life they even begin dragging their third legs. The current paper focuses on characterizing the morphological changes of the motor neurons in the third legs and shows degradation in the regions where MN-I2 (a previously characterized motor neuron) connects to the muscles.

**What are the potential implications of these results for your field of research?**

I hope that the results of the paper can be used to validate our model for use in ALS research as well as encourage other scientists to implement similar models when studying other complex diseases. Eventually, I hope that this model is able to shed light on the pathways involved in the degradation of the motor neurons in humans with ALS and can potentially be used to help find genes and/or drugs that can help rescue the ALS phenotype in the fruit flies.

**What has surprised you the most while conducting your research?**

I think what has surprised me most is just how similar a fruit fly is to a human being, we all share the same building blocks of life DNA, carbohydrates, proteins and since I took my first biology course in high school I have known that. Even still I never really understood just how similar we are until I saw a fruit fly with an ALS-associated mutation drag its back leg. Seeing a fruit fly succumb to a mutation that in humans causes a disease like ALS really shows the ways in which we are the same. Looking at the progressive morphological changes in motor neurons of flies with the ALS associated mutations just cements the similarity and gives me hope that our model can be used to help better understand ALS and maybe one day, beat it.

“…I never really understood just how similar we are until I saw a fruit fly with an ALS-associated mutation drag its back leg.”

**What, in your opinion, are some of the greatest achievements in your field and how has this influenced your research?**

Our research could not have been done without the massive leaps of progress in sequencing technology as well as bioinformatics. The current paper utilizes techniques in sequencing technology in order to help create and validate our model; in addition, bioinformatics tools such as BLAST make finding orthologs to human genes easy to do. This is also not to mention the reduction in sequencing cost can make it so that differential gene expression analysis can performed on the flies in order to get a better idea of the differences in gene expression in the flies with ALS associated mutations and the wild-type flies.

**Figure:**
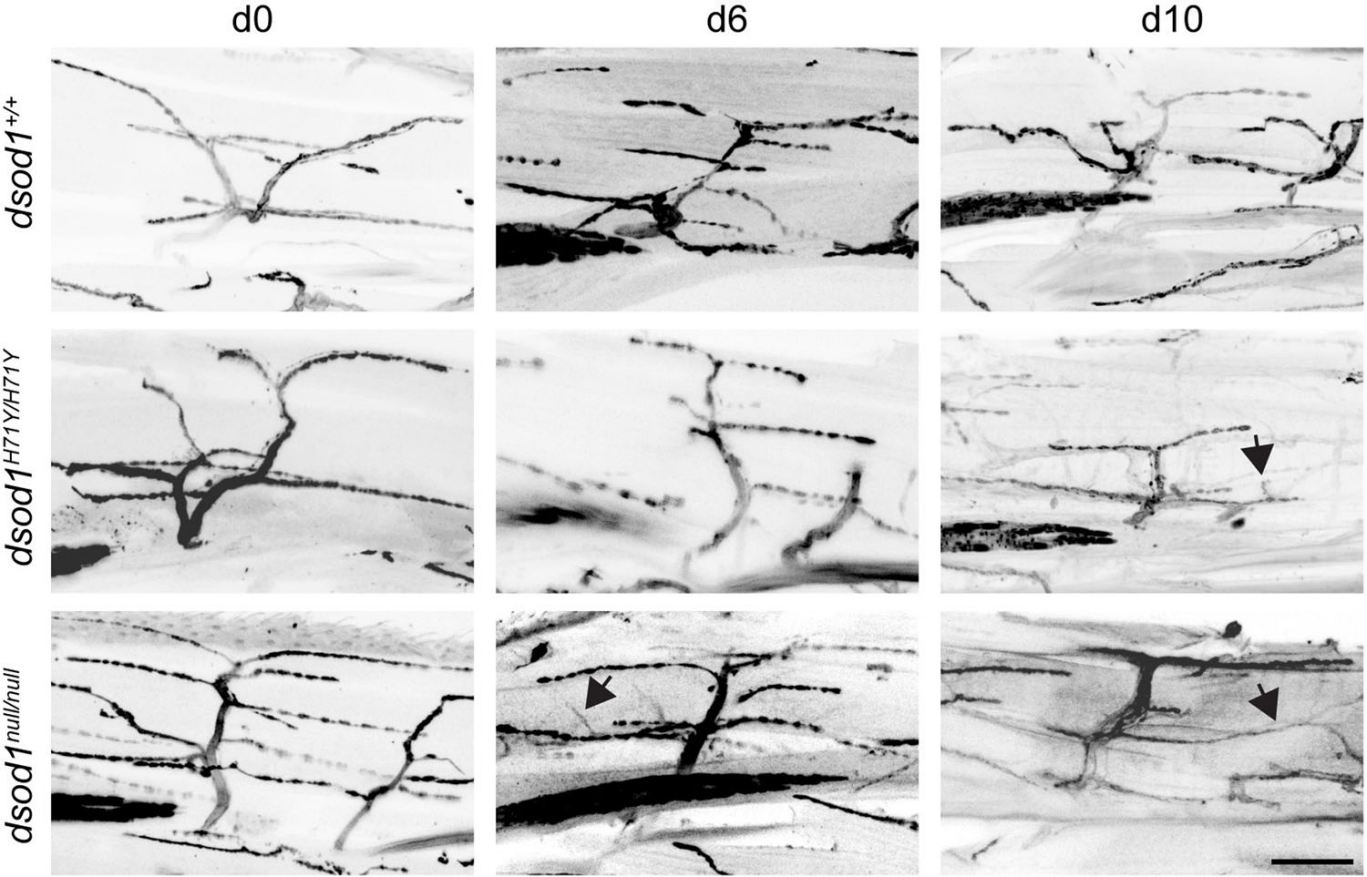
**Retraction of axonal arborization in aged sod mutants.**

**What changes do you think could improve the professional lives of early-career scientists?**

I started research during my second year of university as an undergraduate and I think that this is potentially the most important aspect of improving my professional career. As undergraduate, I had worked on a number of projects in the Stilwell lab before eventually beginning my work on the research that would form the basis for the current paper. Starting research as an undergraduate opened me up to world of academic research early and not only boosted my academic resume but also convinced me that research is what I want to do. For young students who think that a career in science might be something they are interested in I offer the following advice, start early.

“For young students who think that a career in science might be something they are interested in I offer the following advice, start early.”

**What's next for you?**

I hope to go on to a PhD program in computational biology, utilizing the computational techniques that I have picked up while studying pre-eclampsia and pre-term birth such as; machine learning and differential gene expression analysis as well as the wet lab experience that I have gained through studying ALS in the Stilwell lab, to find pathways involved in the pathogenesis of complex diseases as well potentially validate those pathways via animal models.
